# Draft Genome Sequence of *Bacillus methylotrophicus* FKM10, a Plant Growth-Promoting Rhizobacterium Isolated from Apple Rhizosphere

**DOI:** 10.1128/genomeA.01739-15

**Published:** 2016-02-11

**Authors:** Chengqiang Wang, Xiuna Hu, Kai Liu, Qihui Hou, Qianqian Yang, Yanqin Ding, Binghai Du

**Affiliations:** College of Life Sciences, Shandong Agricultural University/Shandong Key Laboratory of Agricultural Microbiology, Taian, China

## Abstract

*Bacillus methylotrophicus* FKM10 is a strain of plant growth-promoting rhizobacterium with antimicrobial activity, which was isolated from apple rhizosphere. Here, we present the genome sequence of *B. methylotrophicus* FKM10. Two scaffolds were finally assembled, and several functional genes related to its antimicrobial activity were discovered.

## GENOME ANNOUNCEMENT

*Bacillus methylotrophicus* is a widespread bacterium in the rhizosphere soil and considered a strain of plant growth-promoting rhizobacterium (PGPR) (1, 2). *B. methylotrophicus* was reported to be able to solubilize *in vitro* insoluble P (3); degrade ferulic acid (4) and hydrocarbon (5); act as an efficient candidate for chromium bioremediation (6); affect rhizosphere enzymes (4); and control many pathogenic microorganisms (7–9) due to the production of secondary metabolites or antibiotics (10). A strain *B. methylotrophicus* FKM10 was isolated from the soil of apple rhizosphere in Shandong, China. It was identified to be a member of PGPR and have antimicrobial activity to some pathogen of soil-borne plant diseases, such as *Fusarium oxysporum*, *F. solani*, and *F. proliferatum*.

Here, we report the draft genome sequence of *B. methylotrophicus* FKM10. The whole genome DNA was extracted and then sequenced using the Illumina HiSeq 2500 platform. Two libraries were constructed with insert sizes of 406 bp and 6,409 bp, respectively. The raw data were filtered and assembled by SOAPdenovo software version 2.04 (11, 12) to generate 2,018 Mb of total clean data. The genome coverage was 513.0×. Two scaffolds were finally obtained, and the total sequence of the obtained genome is 3,928,789 bp, with a G+C content of 46.49%, which is similar to *B. methylotrophicus* YJ11-1-4 (46.4%) (GenBank accession no. NZ_CP011347), *B. methylotrophicus* JS25R (46.39%) (NZ_CP009679), and *B. methylotrophicus* JJ-D34 (46.2%) (NZ_CP011346).

A total of 3,783 genes, 3,621 coding sequences (CDSs), 52 pseudogenes, 82 tRNA genes, and 28 rRNA genes were predicted in the genome of *B. methylotrophicus* FKM10. Two prophage were figured out with an average length of 27,365 bp. The genome sequence was annotated using the NCBI Prokaryotic Genome Annotation Pipeline (PGAP) with the annotation method of GeneMarkS+.

Many genes were predicted to be responsible for the antimicrobial activity of *B. methylotrophicus* FKM10. A large proportion were involved in polyketide synthesis, such as polyketide synthase (PKS) genes (ATE50_08545, ATE50_08550, ATE50_08555, etc.), enoyl-CoA hydratase genes (ATE50_08590, ATE50_10985, and ATE50_10990), polyketide biosynthesis protein gene (ATE50_11020), and polyketide beta-ketoacyl-ACP synthase gene (ATE50_14660). Some other antimicrobial genes were identified, including streptomycin biosynthesis protein StrF gene (ATE50_01775), bacilysin biosynthesis protein BacA or BacE genes (ATE50_02050, ATE50_02080, and ATE50_02070), some kinds of nonribosomal peptide synthetase genes (ATE50_05025, ATE50_10255, ATE50_10260, etc.), and so on. Furthermore, some genes might take charge of the processing or transport of antibiotics, such as the antibiotic biosynthesis monooxygenase gene (ATE50_17270), the antibiotic ABC transporter permease gene (ATE50_02130), and the antibiotic ABC transporter ATP-binding protein genes (ATE50_04260, ATE50_05825, and ATE50_18210).

The genome sequence of *B. methylotrophicus* FKM10 and its genome annotation provide deeper insights into understanding the molecular genetic characteristics of *B. methylotrophicus*, and further understanding the molecular mechanism to control some pathogen of soil-borne plant diseases, which is beneficial for the development of microbial fertilizer or biocontrol agent to improve crop production.

### Nucleotide sequence accession numbers.

The whole-genome shotgun project of *B. methylotrophicus* FKM10 has been deposited at DDBJ/EMBL/GenBank under the accession number LNTG00000000. The version described in the paper is the first version, LNTG01000000.
